# Homologous high-throughput expression and purification of highly conserved *E coli *proteins

**DOI:** 10.1186/1475-2859-6-18

**Published:** 2007-06-06

**Authors:** Asgar Ergin, Konrad Büssow, Joachim Sieper, Andreas Thiel, Rainer Duchmann, Thomas Adam

**Affiliations:** 1 Universitätsmedizin Berlin, Charité, Campus Benjamin Franklin, Hindenburgdamm 30, 12200 Berlin, Germany; 2 Max-Planck-Institut für Molekulare Genetik, Ihnestr. 73, 14195 Berlin, Germany; 3 Deutsches Rheuma-Forschungszentrum, Charitéplatz 1, 10117 Berlin, Germany; 4 Universitätsmedizin Berlin, Charité, Campus Charité Mitte, Dorotheenstr. 96, 10117 Berlin, Germany

## Abstract

**Background:**

Genetic factors and a dysregulated immune response towards commensal bacteria contribute to the pathogenesis of Inflammatory Bowel Disease (IBD). Animal models demonstrated that the normal intestinal flora is crucial for the development of intestinal inflammation. However, due to the complexity of the intestinal flora, it has been difficult to design experiments for detection of proinflammatory bacterial antigen(s) involved in the pathogenesis of the disease. Several studies indicated a potential association of *E. coli *with IBD. In addition, T cell clones of IBD patients were shown to cross react towards antigens from different enteric bacterial species and thus likely responded to conserved bacterial antigens. We therefore chose highly conserved *E. coli *proteins as candidate antigens for abnormal T cell responses in IBD and used high-throughput techniques for cloning, expression and purification under native conditions of a set of 271 conserved *E. coli *proteins for downstream immunologic studies.

**Results:**

As a standardized procedure, genes were PCR amplified and cloned into the expression vector pQTEV2 in order to express proteins N-terminally fused to a seven-histidine-tag. Initial small-scale expression and purification under native conditions by metal chelate affinity chromatography indicated that the vast majority of target proteins were purified in high yields. Targets that revealed low yields after purification probably due to weak solubility were shuttled into Gateway (Invitrogen) destination vectors in order to enhance solubility by N-terminal fusion of maltose binding protein (MBP), N-utilizing substance A (NusA), or glutathione S-transferase (GST) to the target protein. In addition, recombinant proteins were treated with polymyxin B coated magnetic beads in order to remove lipopolysaccharide (LPS). Thus, 73% of the targeted proteins could be expressed and purified in large-scale to give soluble proteins in the range of 500 μg.

**Conclusion:**

Here, we report a cost-efficient procedure to produce around 200 soluble recombinant *E. coli *proteins in large-scale, including removal of LPS by polymyxin B coated beads for subsequent use of the proteins in downstream immunological studies.

## Background

Inflammatory Bowel Disease (IBD) comprises Crohn's disease (CD) and ulcerative colitis (UC). CD is characterized by chronic granulomatous inflammation throughout the entire gastrointestinal tract, with the terminal ileum mainly affected. In UC, chronic inflammation is limited to the colorectum, continuous and without granuloma formation [1]. So far, the etiology of IBD is not fully understood. Genetic factors, environmental factors and a dysregulated immune response towards commensal bacteria contribute to the pathogenesis [1].

In CD, exposition of the mucosal immune system to the intestinal flora may result in the production of proinflammatory cytokines [2]. Most animal models of IBD are characterized by increased T helper 1 (Th1) cytokine production [3]. Interferon-γ (IFN-γ) was shown to be causatively involved in experimental IBD [4]. In animal models of IBD, the normal enteric bacterial flora plays a key role in the development of the disease [1,3,5]. DNA of *E. coli *was detected in 80% of CD granulomas by Laser Capture Microdissection and PCR [6]. Another study demonstrated the presence of *E. coli*, *Listeria*, and *Streptococcus *antigens within macrophages of CD patients by immunohistochemistry [7]. *E. coli *is predominant in ileal mucosa [8,9]. In addition, an adherent-invasive *E. coli *(AIEC) strain was isolated from a chronic lesion of a CD patient. AIEC survived and replicated in the host cell cytoplasm after lysis of the endocytic vacuole [10] and were shown to survive and replicate within macrophages [11]. AIEC are associated with inflammatory lesions of CD patients [12]. Finally, Gram-negative bacteria, i.e *E. coli*, were shown to aggravate Th1 type immunopathology in an animal model for small intestinal inflammation [13].

In IBD, Duchmann et al. demonstrated T cell reactivity towards bacterial antigens shared by different *Enterobacteriaceae*, including *E. coli *[14]. Thus, conserved antigens of intestinal bacteria could drive chronic inflammation directly or via induction of autoimmunity. Therefore, we aimed at a systematical study of a potential role of conserved *E. coli *proteins in the pathogenesis of IBD.

The most conserved protein functions are represented in all three biological kingdoms, Archaea, Prokarya, and Eukarya. Thus, we first chose a set of *E. coli *proteins hypothetically inherited by the Last Universal Common Ancestor (LUCA) [15] of the three kingdoms. As a second set we identified additional conserved proteins between *E. coli *and humans that were not included in the LUCA set of proteins. In general, these highly conserved proteins are not represented in Archaea and therefore not included in the LUCA proteins. These proteins are referred to as *E. coli*-Human-Homologues (ECHH). Since most of the highly conserved protein functions addressed in this study also are represented in humans, T cell reactivity towards these proteins could give a hint to potentially autoreactive human antigens in IBD.

In this project, we aimed at the production of purified ECHH and LUCA proteins represented in *E. coli *at amounts of around 500 μg, suitable for downstream whole blood T cell stimulation assays.

Cloning steps such as amplification of target genes with polymerase chain reaction (PCR), DNA purification, digestion of target genes with restriction enzymes, vector ligation, and transformation of chemically competent *E. coli *cells were performed in a 96-well microtitre plate format. In order to identify clones that provided sufficient amounts of proteins, a high-throughput purification method using a pipetting robot was applied as recently established in the Protein Structure Factory [16]. Initially, clones were subjected to small-scale expression. Histidine-tagged (His-tag) proteins were purified under native conditions *via *affinity chromatography to a nickel chelate matrix. After cell lysis, soluble proteins were expected to bind to the matrix. Elution of bound proteins into a denaturing buffer and SDS-PAGE analysis identified proteins that were purified in sufficient yields. Suitable expression clones were then used for large-scale protein expression and purification to obtain a minimum of 500 μg per protein for downstream immunological studies. During lysis of bacterial cells, lipopolysaccharide (LPS), a component of the outer membrane of Gram-negative bacteria, can be released. LPS is a strong activator of the innate immune system *via *Toll-like receptor 4 (TLR-4) [17]. Since LPS could interfere with downstream immunological applications [18-20], such as *in vitro *stimulation of whole blood or peripheral blood mononuclear cells (PBMCs), we decided to include a step for LPS removal in the protocol for large-scale production of proteins. A variety of ligands has been used for removal of LPS [21], including polymyxin B which was shown to neutralize LPS activity in PBMCs [22]. To remove LPS from protein solutions, we used a rapid, automatable procedure based on polymyxin B coated magnetic beads.

Herein, we report on cloning, large-scale expression and purification of 197 of the most conserved *E. coli *proteins.

## Results

### LUCA proteins

**Figure 1:**
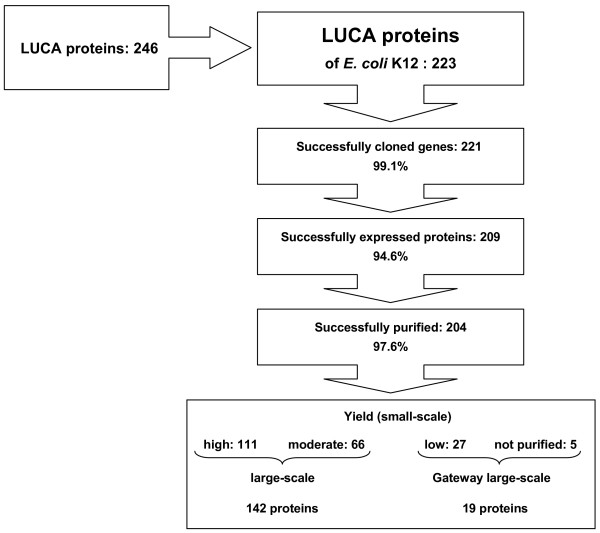
**Step-by-step efficiency of cloning, small-scale protein expression and purification of *E. coli*LUCA proteins. **177 purified proteins with high and moderate yields were subjected to large-scale expression. 142 proteins could be purified with a minimum of 500 μg. 32 target genes were subjected to Gateway recombination cloning in order to express fusion proteins with MBP-His_7_, GST-His_7_, and NusA-His_6_, respectively. Large-scale expression revealed 19 purified proteins fused to MBP-His_7 _with a minimum of 500 μg.

**Figure 2:**
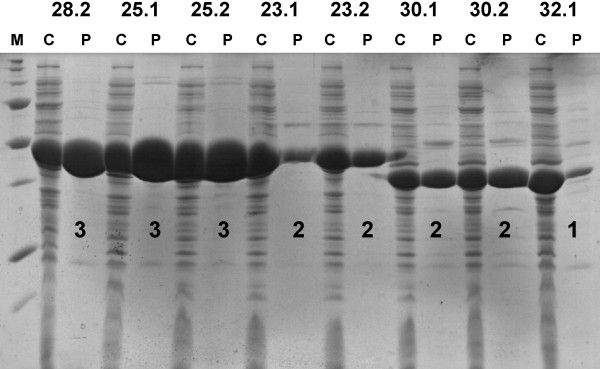
**SDS-PAGE analysis of cell extracts after lysis of bacteria and purified proteins. **Cell lysates (C) and purified proteins (P) were mixed with 4xSDS-PAGE sample buffer. Protein bands were visualized by Coomassie staining. Yields of purified proteins were classified as indicated with the numbers below the protein bands (3: high, 2: moderate1: low). Numbers on top of the panel designate proteins as given in additional file 1. Note that only one clone per protein was chosen for large-scale purification.

We were able to identify in the *E. coli *K12 genome 223 of 246 LUCA proteins described by Kyrpides et al. [15](see additional file 1: SummaryOfResults.xls). Of 223 LUCA genes represented in *E. coli*, we successfully cloned 221 (99.1%) into pQTEV2 (Fig 1). In small-scale, expression of 209 His-tagged proteins (94.6%) was detected in the cell extracts. Of these 209 proteins, 5 proteins could not be purified under native conditions with Ni-NTA agarose (proteins 198, 199, 205, 208, 209). 204 proteins (97.6%) could be purified. To obtain maximum amounts of protein, 161 proteins were expressed at 37°C and 43 proteins at 25°C (data not shown). 111 proteins (54.4%) were purified in high yields, 66 (32.4%) in moderate, and 27 (13.2%) in low yields. The estimation of protein yields is illustrated in Fig. 2. Together with the genes coding for the 5 proteins that were not purified, the genes of 27 proteins with low yields were subjected to Gateway (Invitrogen) recombination cloning in order to improve solubility by fusing the target proteins to MBP-His_7_, GST-His_7_, and NusA-His_6_.

### ECHH proteins

**Figure 3:**
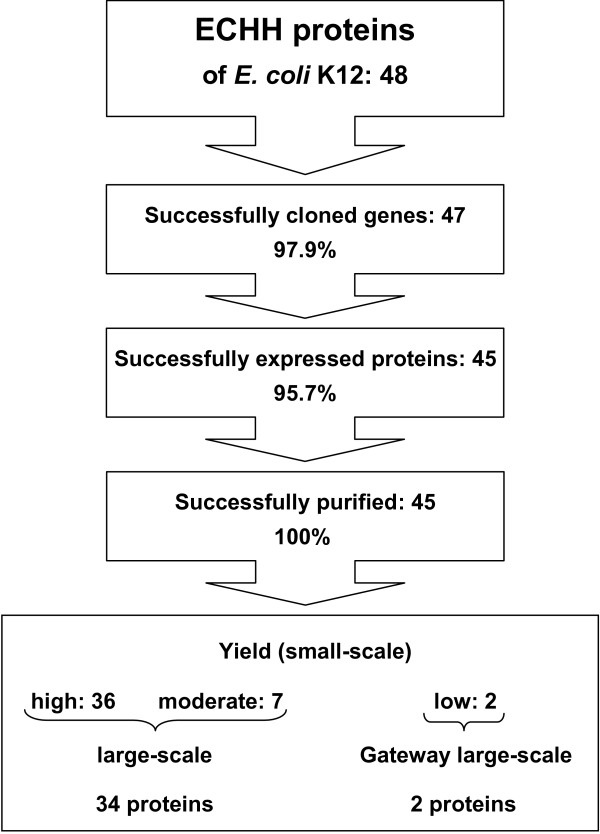
**Step-by-step efficiency of cloning, small-scale protein expression and purification of ECHH proteins. **43 purified proteins with high and moderate yields were subjected to large-scale expression. 34 proteins could be purified with a minimum of 500 μg. Two target proteins were subjected to Gateway recombination cloning in order to express fusion proteins with MBP-His_7_, GST-His_7_, and NusA-His_6_, respectively. Both proteins fused to MBP-His_7 _were purified with a minimum of 500 μg.

Of 48 ECHH genes, we successfully cloned 47 (97.9%) into pQTEV2 (Fig 3). Expression of 45 His-tagged proteins (95.7%) was detected in the bacterial lysate. To obtain maximum amounts of protein, 40 proteins were expressed at 37°C and 5 proteins at 25°C (data not shown). All of these 45 proteins could be purified with Ni-NTA agarose. 36 proteins (80%) were purified in high yields, 7 (15.5%) in moderate, and two (4.5%) in low yields. These two genes were also subjected to Gateway recombination cloning.

In total, 14 LUCA or ECHH proteins could not be expressed in *E. coli *SCS1 (see additional file 1: SummaryOfResults.xls). Some proteins could not be expressed at all. Other proteins could be expressed, but revealed low yields in small-scale purification or could not be purified. The distribution of these proteins into functional groups is illustrated in Table 1. Interestingly, 53% of transport proteins present in either protein sets were not expressed. 33% of ribosomal proteins were purified with low yields. 187 enzymes (87.4%), 15 ribosomal proteins (62.5%), but only 3 transport proteins (21.4%) were purified in soluble form with at least moderate yields.

**Table 1:** Distribution of "difficult" LUCA and ECHH proteins among functional groups of proteins

	**enzymes **(including putative) (214)	**ribosomal proteins **(24)	**transport proteins **(14)	**putative structure proteins **(3)
not expressed	6	-	8	-
not purified	3	1	1	-
purified in low yields	18	8	2	1

Of 268 LUCA and ECHH proteins, 14 proteins were not expressed in small-scale. 34 proteins were expressed. 5 of those proteins could not be purified and 29 were purified in low yields. Numbers in brackets give the total number of proteins in respective functional group. Note that all 14 non-expressed proteins contain at least 2 transmembrane helices.

### Gateway cloning

Corresponding genes of the 34 proteins that were not purified or displayed low yields under native conditions were sub-cloned into Gateway destination vectors in order to fuse the target proteins to MBP-His_7_, GST-His_7_, and NusA-His_6_, respectively. The resulting 102 fusion proteins were overexpressed in small-scale and purified by His-tag affinity to Ni-NTA agarose. Per target protein, the fusion partner was determined that most efficiently improved the yield after purification. Table 2 illustrates yields of 34 target proteins fused to the His_7_-tag and to MBP-His_7_, GST-His_7_, or NusA-His_6_. Yields of 30 target proteins improved with fusion to MBP-His_7 _(80%), NusA-His_6 _(16.7%) or GST-His_7 _(3.3%). Yields of 4 proteins including two membrane proteins were not affected.

**Table 2:** Gateway recombination cloning of 34 LUCA and ECHH genes

**No.**	**Protein**	**Yield with His**_7_**-tag**	**Fusion with**	**Yield**
157	aspartokinase I; homoserine dehydrogenase I	1	MBP	3
145	acetolactate synthase III small subunit	1	MBP	3
203	thiogalactoside acetyltransferase	1	MBP	2
200	4-hydroxy-2-ketovalerate aldolase	1	MBP	2
258	putative ATPase	1	MBP	3
204	anthranilate synthase component II	1	MBP	2
155	putative ATP-binding component of a transport system	1	MBP	3
201	flagellum-specific ATP synthase	1	MBP	2
259	fused enoyl-CoA hydratase and epimerase	1	MBP	2
156	FFh	1	MBP	2
146	30S ribosomal subunit protein S11	1	MBP	2
147	30S ribosomal subunit protein S13	1	MBP	3
148	50S ribosomal subunit protein L14	1	MBP	3
149	50S ribosomal subunit protein L22	1	MBP	3
151	50S ribosomal subunit protein L23	1	MBP	2
152	30S ribosomal subunit protein S12	1	MBP	2
150	50S ribosomal subunit protein L2	1	MBP	3
154	50S ribosomal subunit protein L1	1	MBP	3
153	biotin- [acetyl-CoA carboxylase] holoenzyme synthetase	1	MBP	3
159	RNA polymerase beta prime subunit	1	MBP	2
143	predicted acyltransferase with acyl-CoA N-acyltransferase	1	MBP	3
144	putative proteoglycan	1	MBP	2
158	endonuclease III	1	MBP	2
161	dihydroxyacid dehydratase	1	MBP	3
202	part of formate-dependent nitrite reductase complex	1	NusA	2
206	conserved protein, member of DEAD box family	1	NusA	2
198	50S ribosomal subunit protein L5	0	NusA	2
205	O-6-alkylguanine-DNA/cysteine-protein methyltransferase	0	NusA	1
199	acetylornithine delta-aminotransferase	0	NusA	1
197	inducible ATP-independent RNA helicase	1	GST	2
160	glutamate synthase large subunit	1		-
207	carbamoyl-phosphate synthase large subunit	1		-
208	ATP-binding transport protein (membrane protein)	0		-
209	Mg2+ transport ATPase, P-type 1 (membrane protein)	0		-

Yields of purified proteins were analyzed by SDS-PAGE before recombination (proteins fused to His_7_-tag) and after recombination (each target protein fused to MBP-His_7_, GST-His_7_, or NusA-His_6_, respectively). The fusion partner that most efficiently improved protein yield is shown. With regard to yield, proteins were attributed to one of four categories: 3 (high), 2 (moderate), 1 (low), 0 (not purified).

### LPS removal

**Figure 4:**
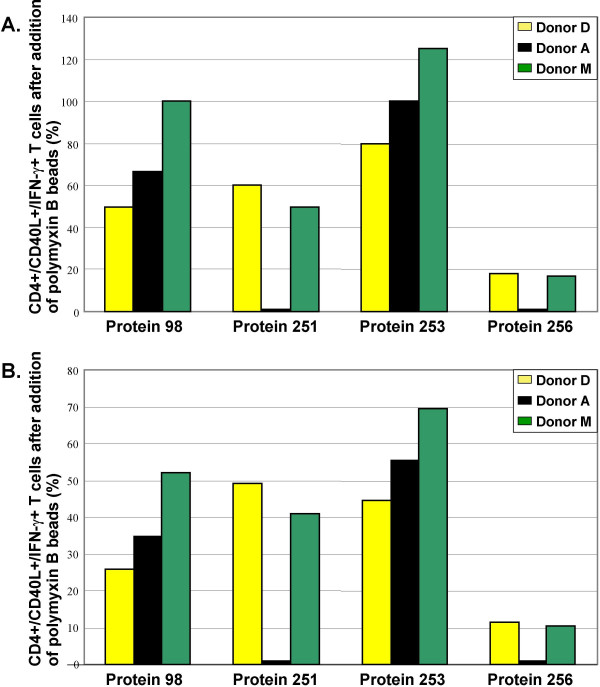
**Effect of LPS removal procedure on T cell stimulation. A. **Equal amounts of proteins: Four proteins were treated with LPS removal beads. Whole blood of 3 healthy donors was stimulated with 5 μg/ml of treated or non-treated protein preparations. After fixation and permeabilization, leukocytes were stained with antibodies against CD4, CD40L, and IFN-γ. The stained cells were measured by FACS. CD4+ T cells were gated electronically. Frequencies of double-positive T cells stimulated with treated preparations were related to frequencies obtained after stimulation with untreated proteins. The latter frequencies were standardized (set to 100%). **B. **Equal volumes: The results of the experiment given in A. are displayed considering equal volumes of treated and untreated protein preparations, respectively. Representative results from 3 of 8 individuals tested.

Four *E. coli *proteins were treated with polymyxin B coated magnetic beads in order to analyze efficiency of this protocol to reduce potential LPS related stimulation of CD4+ T cells. We compared the frequencies of CD40L+/IFN-γ+ T cells resulting from whole blood stimulations with protein solutions before vs. after treatment with polymyxin B beads. Equal amounts of proteins were applied, i.e. 5 μg per ml blood. Treatment of proteins 98, 251, and 256 with LPS removal beads reduced the frequency of stimulated CD4+ T cells. Except for protein 98, analyzed in blood of donor M, all donors showed decreased frequencies. Especially, stimulations with protein 256 revealed a drastic decrease in all donors. In contrast, treatment of protein 253 with LPS removal beads did not reduce the frequency of stimulated CD4+ T cells. In donor M, the frequency of double-positive cells stimulated with protein 253 even increased (Fig 4A). Since we observed variable loss of protein after incubation with LPS removal beads, different volumes of protein solutions had to be taken in subsequent immunological assays to provide 5 μg of protein per assay. This may have increased the amount of other stimulatory bacterial contaminants, such as muramyl dipeptide [23] and lipoproteins [24]. Therefore, an additional analysis of the frequencies after stimulation with equal volumes of treated vs. untreated protein solutions was done (Fig. 4B). Without exception, treatment with LPS removal beads resulted in decreased frequencies.

In addition, LPS contents of protein solutions 251 and 96 were quantified with the *Limulus *amoebocyte lysate (LAL) test. Both protein solutions were treated with LPS removal beads. The LPS content in the protein solution 96 was below the detection limit of 0.05 EU/ml (5 pg/ml), whereas 0.08 EU/ml (8 pg/ml) was measured in protein solution 251.

Next, we quantified protein loss by the LPS removal procedure. For this, protein concentrations of 34 different protein preparations were determined before and after treatment with LPS removal beads. More than 61% of the analyzed proteins revealed a protein loss less than 50%. Only 5.9% of the proteins revealed a protein loss of 80–90% (Table 3). Since only few proteins showed major loss by the LPS removal procedure, we applied the procedure to all protein preparations of this study.

**Table 3:** Protein recovery of 34 *E. coli *proteins after incubation with LPS removal beads

**Protein recovery (%)**	**Number of proteins**
10 – 20	2
21 – 50	11
51 – 90	7
> 90	14

After treatment with LPS removal beads, protein recovery was determined and related to protein content before treatment (in %).

### Large-scale expression and purification

In order to produce a minimum of 500 μg of purified soluble protein, expression culture volumes were scaled up. By small-scale expression and purification, we obtained 177 LUCA proteins with high and moderate yields (Fig 1). However, in 35 proteins we did not achieve the minimum of 500 μg of purified protein. 26 of 32 Gateway LUCA fusion proteins revealed high or moderate yields when expressed in small-scale. Of these, 19 were expressed and purified in large-scale as fusions to MBP-His_7 _(Fig 1). Of 43 ECHH proteins with high and moderate yields when expressed in small-scale, 9 proteins could not be produced in sufficient amounts. In addition, we obtained 2 ECHH proteins from Gateway fusions to MBP-His_7_. Thus, we were able to produce 36 ECHH proteins with a minimum of 500 μg (Fig 3).

### Codon usage

**Figure 5:**
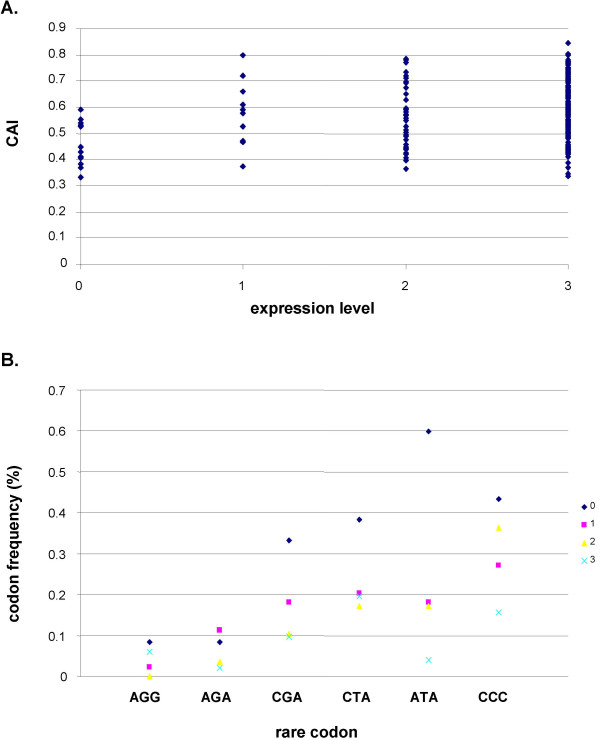
**Codon usage. **A. Global analysis: The codon adaptation index (CAI) was determined for each protein, sorted by the level of expression. B. Analysis of 6 individual rare codons. 52 poteins were included: All 14 non-expressing proteins, all 10 proteins of low expression level, and a random choice of 14 proteins each of moderately and highly expressed proteins. The graph gives codon frequencies for each of the six codons in the total of codons in proteins of identical level of expression (no of codon X/total no of codons in proteins of expression group).

In order to correlate codon usage of proteins with success of expression, the codon adaptation index (CAI)[25], a global indicator which compares codon usage of individual proteins with codon usage of a set of reference proteins, was determined for all proteins. As shown in Fig 5A., all proteins with a CAI of > 0.6 could be overexpressed in our system. In addition, we studied individual codons most rarely used in *E. coli *in a representative set of proteins. This analysis revealed that some rare codons are overrepresented in non-expressed proteins (Fig 5B.). Of particular interest might be the rare isoleucine codon ATA which occured 3 times or 15 times more frequently in non-expressed proteins than in weakly or highly expressed proteins, respectively (Fig 5B).

### Transmembrane helices

The 48 proteins that could not be expressed or yielded no protein or insufficient quantities of protein after purification as non-fusion proteins (see Table 1) were subjected to an *in-silico *analysis for the presence of predicted transmembrane domains. In addition, an arbitrarily chosen protein was included in this study. The results of this analysis are given in the additional file 1 (SummaryOfResults.xls). All 14 non-expressed proteins contained at least 2 predicted transmembrane helices (proteins 210–221, 269, 270). The two proteins that could be expressed but not be purified even as fusion proteins contained 5 or 7 predicted transmembrane helices, respectively (proteins 208, 209). On the other hand, at least 3 proteins containing 1, 2, or 8 predicted transmembrane helices, respectively, could be expressed and purified in sufficient quantities using our approach (proteins 202, 163, 258).

## Discussion

Our aim was the homologous expression and purification of a large number of the most conserved *E. coli *proteins. We aimed at producing a minimum amount of 500 μg per protein for downstream immunological studies. For this, we cloned target genes into the expression vector pQTEV2, which provides a His_7_-tag. Overexpression and purification by nickel affinity chromatography was performed in small-scale in order to identify the clones that yielded soluble proteins. Proteins that were not purified or revealed low yields were fused to fusion partners to enhance solubility (Gateway recombination cloning). We successfully amplified 99% of the target genes. Our amplification results confirm recently published data targeting bacterial genes [26-28]. Our PCR products were digested with BamHI and NotI or alternative enzymes in a few cases. All PCR products were successfully cloned into the expression vector pQTEV2. Subsequently, 94% of the selected *E. coli *proteins could be expressed as His_7_-tag fusions in small-scale. The rate of expressed proteins was as described [27]. Small-scale expression of the *E. coli *proteins showed that 87% of the selected *E. coli *proteins were expressed as His_6_-tag fusions using the Gateway system [27]. In this study, different *E. coli *host strains were used in order to optimize expression efficiency. Proteins of the eukaryotic organism *Caenorhabditis elegans *were also expressed in *E. coli*. 10,167 ORFs were cloned into a Gateway vector. Two different strains were used as hosts. However, only 48% of the proteins could be expressed in small-scale [29]. In experiments where human proteins were expressed in *E. coli*, only 16% of these proteins were expressed in soluble form [30]. Similar results were obtained with *C. elegans *proteins: 15% of the selected proteins were soluble [29]. Small numbers of soluble eukaryotic proteins are not surprising and could reflect problems with different codon usage or lacking posttranslational modifications in *E. coli*. In small-scale, 92% of our conserved *E. coli *proteins were found to be expressed in soluble form in the host cell. This high rate may result from our selection of highly conserved proteins. In a different set of *E. coli *proteins, only 60% of target proteins were expressed in soluble form [27]. A similar observation was made with soluble expression of *B. anthracis *proteins in *E. coli *with 86% of conserved proteins but only 69% of non-conserved proteins [31]. Our strategy to clone target genes with standard molecular techniques into pQTEV2 in order to generate His_7_-tagged proteins in the *E. coli *host strain SCS1, revealed a large number of purified proteins. In cases where proteins were purified with low yields or were not purified, we utilized the Gateway recombination technology in order to fuse these proteins with MBP-His_7_, GST-His_7_, or NusA-His_6_. Fusion of target proteins to these partners was expected to improve solubility [32]. Indeed, with regard to our *E. coli *proteins, yields of most target proteins improved when fused to MBP-His_7 _(Table 2). This finding confirms data on 6 target proteins of various origins [33]. Shih et al. [34] found that 60% of analyzed eukaryotic proteins were expressed in soluble form when fused to MBP or NusA. 38% of the target proteins were expressed in soluble form when fused to GST. In our study, GST and NusA were less efficient in improving the yields of purified proteins. This could be due to the different sets of proteins studied (eukaryotic vs. prokaryotic proteins).

Small-scale expression was performed with two clones per transformation. Using this strategy, we could determine a minimum of one clone per transformation that expressed protein, except for 18 genes. In the latter cases, we tested additional two clones for protein expression and tried small-scale purification of soluble protein. This approach enabled us to identify two more proteins expressed in soluble form. Concerning the remaining 16 proteins, we repeated the cloning procedure, starting with PCR of target genes. After the second round of cloning, again two more proteins expressed in soluble form were identified. Finally, 14 proteins could not be expressed, even after a third round of cloning and expression. These proteins consisted of membrane proteins containing at least two transmembrane helices. Overexpression and purification of membrane proteins was reported to be difficult [35]. Due to potential formation of inclusion bodies, purification under native conditions may not be the appropriate method for these proteins. Instead, purification under denaturing conditions might result in sufficient protein yields in a view of these proteins. Furthermore, non-expressing proteins showed higher frequencies of rare codons. Therefore, a number of the proteins that failed to be expressed in our system could possibly be made in host strains overexpressing rare t-RNAs. Alternatively, such rare codons could be mutated in order to increase efficiency of expression.

During growth, division, and lysis of *E. coli *cells, LPS is released [21]. Because the proteins were produced for immunological assays, such as whole blood stimulations, the removal of LPS was an important task. At neutrality, LPS is negatively charged. One potent LPS ligand is polymyxin B. At low ionic strength, it is positively charged at the amino groups. Polymyxin B binds LPS mainly through hydrophobic interactions with Lipid A and electrostatic interactions. Electrostatic interactions between the negatively charged LPS and polymyxin B are supposed to be stronger than between proteins and polymyxin B. In addition, charged and hydrophobic groups are fixed in proteins due to their globular structure. Still, binding of negatively charged proteins to polymyxin B is possible, thus causing protein loss [21]. Analysis of whole blood stimulations showed that polymyxin B treated proteins revealed less background stimulation of CD4+ T cells, compared with equal amounts of untreated proteins (Fig. 4A). When equal volumes of treated or untreated proteins were analyzed, reduced backgrounds were observed (Fig. 4B). Provided that the proteins did not elicit antigen-specific T cell responses, this reducing effect may have been due to removal of LPS. Otherwise, this may reflect less stimulatory effect by the proteins because of their dilution. Either way, it is most likely that other bacterial contaminants did not have a major stimulatory influence. In order to determine LPS concentrations in two protein solutions that were treated with LPS removal beads, the LAL test was performed. Both protein solutions contained less than 9 pg/ml LPS. Nakagawa et al. [36] stimulated whole blood with endotoxin (LPS) in order to determine the minimum amount of LPS to induce cytokine secretion in monocytes. The determined detection limit for both, TNF-α and IL-6 secretion was 14 pg/ml, whereas a minimum LPS concentration of 100 pg/ml was required to detect LPS-induced IL-1 secretion. Clearly, LPS concentrations of both protein solutions were below these critical concentrations.

In the large-scale expression procedure, we observed that 52 proteins were not recovered as expected, although small-scale expression and purification indicated moderate or high yields. Precipitation was often observed immediately after affinity chromatography of these proteins. This was not observed in small-scale purification, since the proteins were solubilized directly after elution with 4xSDS sample buffer, whereas in large-scale purifications, proteins were subjected to additional purification steps, i.e. buffer exchange and LPS removal. In addition, precipitation may be explained by higher concentrations of proteins in large-scale preparations.

## Conclusion

Taken together, we obtained 73% of the selected *E. coli *proteins with sufficient amounts after purification. Our strategy combining standard restriction cloning with the Gateway recombination system proved to be a reliable and efficient approach to achieve our aim.

## Methods

### Identification of *E. coli *K12 targets

*E. coli *K12 representatives of the LUCA protein functions as described by Kyrpides et al. [15] were identified in the NCBI protein database. In addition, the protein databases Biocyc.org and Brenda were screened for LUCA proteins present in the *E. coli *genome. Moreover, the 246 LUCA proteins represented in *M. jannaschii *were used as queries in BlastP searches against the *E. coli *K12 protein sequences. By these methods, we identified 223 LUCA genes in the *E. coli *K12 genome. Using the BlastP algorithm [37] for comparison of *E. coli *K12 proteins with human protein equivalents, *E. coli *proteins with highest homologies to human proteins were identified. LUCA proteins were excluded from this list. Thus, 48 ECHH proteins with highest homologies to human proteins and not represented in the LUCA set of proteins were identified.

### Construction of vectors

#### Expression vector pQTEV2

**Figure 6:**
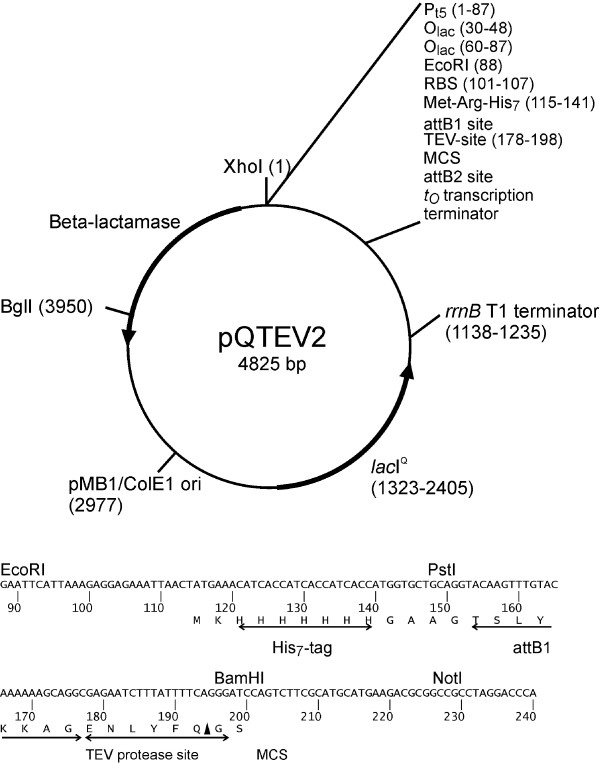
**Features of expression vector pQTEV2**. The vector was used for expression of His7-tagged proteins. Due to the presence of attB1/2 sites, pQTEV2 is suitable for Gateway recombination cloning.

pQTEV2 (Fig 6) [GenBank:EF429248] was derived from pQTEV [30]. A DNA fragment with EcoRI and HindIII overhangs and coding sequences of His_7_-tag, Gateway attB recombination sites, and TEV protease site was cloned directionally into pQTEV. The expression vector pQTEV2 was propagated in electro-competent *E. coli *XL1-blue cells and the correct DNA sequence was verified by DNA sequencing.

#### Gateway destination vectors pD-GEX1, pD-MAL1, and pD-Nus1

Destination vector pD-GEX1 [GenBank:EF431916] was derived from vector pGEX-5X-1 (GE healthcare). A DNA fragment with BamHI and NotI overhangs and coding sequences for the His_7_-tag and the SmaI restriction site was cloned directionally into pGEX-5X-1 (5'-BamHI-CATCACCATCACCATCACCATTCCCGGGC-NotI-3'). The resulting vector was digested with SmaI, followed by the ligation with the Gateway cassette RfC.1 to clone pD-GEX1.

Destination vector pD-Mal1 [GenBank:EF431917] was derived from pMAL-c2X (NEB). A DNA fragment with SacI and HindIII overhangs and coding sequences for the His_7_-tag and the SmaI restriction site was cloned directionally into pMAL-c2X (5'-SacI-CGCATCACCATCACCATCACCATTCCCGGGA-HindIII-3'). The vector was digested with SmaI, followed by ligation-cloning of Gateway cassette RfC.1 into the vector.

Destination vector pD-Nus1 [GenBank:EF431918] was derived from pET-44a (Novagen). Vector pET-44a was digested with SacII and XhoI. The overhangs were removed by treatment with T4 DNA polymerase (NEB), followed by ligation-cloning of Gateway cassette RfA into the vector. Note that this vector encodes two His_6_-tags.

All destination vectors were propagated in chemically competent *E. coli *DB3.1 cells. Correct DNA sequences of the destination vectors were verified by DNA sequencing. Expression of proteins in pD-Mal1, pD-GEX1, or pD-Nus1 resulted in MBP-His_7_-tagged, GST-His_7_-tagged, or NusA-His_6_-tagged fusion proteins, respectively.

### *E. coli *host strains

Competent *E. coli *cells were prepared with CaCl_2 _and MnCl_2 _as described previously [38]. The competent cells were frozen in liquid nitrogen and stored at -80°C. Electro-competent *E. coli *cells were prepared as described previously [39]. The competent cells were frozen in liquid nitrogen and stored at -80°C.

### Cloning of *E. coli *K12 genes into pQTEV2

Our standard procedure employed PCR cloning of *E. coli *genes [30] into pQTEV2 (Fig 6) using restriction sites BamHI and NotI and a genomic DNA preparation from *E. coli *K12 from the German strain collection (DSMZ 5695). In case of intragenic BamHI sites, this enzyme was replaced by BglII. A second choice was use of type II enzymes BpiI or Eco31I. PCR primers were delivered in 96-well microplate format (Eurogentec). 10 μM stocks of forward and reverse PCR primers were rearranged by a Speedy pipetting robot (Zinsser) in corresponding plate positions of two polystyrene microtitre plates [30]. PCR reactions were performed with Expand High Fidelity PCR kit (Roche Applied Science) according to the manufacturer's instructions. The PCR products were analyzed and quantified by agarose gel electrophoresis. Subsequently, the PCR products were purified with magnetic beads (Genopure ds kit, Bruker) according to the manufacturer's instructions. The PCR products were digested o/n with restriction enzymes with 10 units of each enzyme per reaction. After an additional purification step with magnetic beads, ligation reactions were set up taking into account visual estimates of PCR product concentrations. PCR product and water were added to make 6.5 μl in a well of a Thermowell 96-well plate, followed by the addition of 2 μl of linearized pQTEV2 (16 ng), 0.5 μl T4 DNA ligase (400 units/μl, NEB), and 1 μl 10× ligase buffer (NEB). The plate was covered with a sealing sheet and the samples were incubated at 20°C for 1 h, followed by heat inactivation (65°C, 20 min). Chemically competent *E. coli *SCS1 cells [30] were transformed with 5 μl of the ligation reaction in a Thermowell 96-well plate using standard heat shock procedure (42°C, 45 sec). Cells were recovered for 30 min at 37°C with 1 ml of 2xYT broth [16], supplemented with 20 mM MgCl_2 _and 20 mM glucose. The cultures were plated on individual 2xYT agar plates, containing 100 μl/ml ampicillin and were incubated o/n at 37°C. Six colonies per transformation were picked with sterile tooth picks into individual wells of a polystyrene microtitre plate, containing 200 μl of stock medium: 2xYT broth, 1xHMFM, 100 μl/ml ampicillin, and 2% glucose (10xHMFM: solution "a": 5 mM MgSO_4_.4H_2_0, 20 mM tri-Sodium citrate.2H_2_O, 85 mM (NH_4_)_2_SO_4_, 45% glycerol; solution "b": 0.66 M KH_2_PO_4_, 1.3 M K_2_HPO_4_; four parts of solution "a" were combined with one part of solution "b"). The plate was incubated o/n at 37°C, sealed and stored at -80°C. To identify successfully cloned inserts, 6 clones per transformation were screened by colony PCR with primers pQE65 (5'-TGAGCGGATAACAATTTCACACAG-3') and pQE276 (5'-GGCAACCGAGCGTTCTGAAC-3') which bind to internal vector sequences adjacent to the multiple cloning site. After analysis of the PCR reactions on agarose gels, two positive clones per transformation were sub-cultured for protein expression in individual wells of a polystyrene microtitre plate, containing 200 μl of stock medium.

### Gateway cloning

We utilized the Gateway (Invitrogen) recombination technology [40] to fuse the target protein with fusion partners, such as MBP, GST, or NusA. These fusion partners may enhance solubility of target proteins [32]. We therefore constructed three Gateway destination vectors for expression of target proteins fused to MBP-His_7_, GST-His_7_, and NusA-His_6 _(pD-MAL1, pD-GEX1, and pD-Nus1, respectively). All three destination vectors contained the sequence coding for a His-tag, enabling purification of either protein fusion by His-tag affinity to Ni-NTA agarose. In order to create entry clones, we performed BP recombination reactions between pQTEV2 containing the gene of interest and the Gateway donor vector pDONR221, followed by the transformation of *E. coli *XL1-blue cells, according to the manufacturer's instructions (Invitrogen). Positive entry clones were confirmed by colony PCR screening with primers M13Forward and M13Reverse which bind to internal vector sequences adjacent to the att sites. In the LR reaction, plasmid preparations of entry clones were incubated with the destination vectors pD-MAL1, pD-GEX1, and pD-Nus1, respectively, in order to generate *E. coli *SCS1 expression clones for MBP-His_7 _or GST-His_7 _fusion proteins. Electro-competent *E. coli *BL21 (DE3) cells were transformed with LR reactions of pD-Nus1. Positive expression clones were confirmed by colony PCR screening. pD-MAL1 constructs were screened with primers M13Forward (5'-Gcggatcctacctgacgcttt-3') and malE (5'-GGTCGTCAGACTGTCGATGAAGCC-3'). pD-GEX1 constructs were screened with primers pGEX5' (5'-GGGCTGGCAAGCCACGTTTGGTG-3') and pGEX3' (5'-CCGGGAGCTGCATGTGTCAGAGG-3'). pD-Nus1 constructs were screened with primers Nus-tag (5'-AAGCCGGAGCACTGATTATGG-3') and Colidown (5'-TTCACTTCTGAGTTCGGCATGG-3').

### Characterization of expression clones by small-scale protein expression and purification

To identify clones that provided sufficient amounts of His-tagged proteins, small-scale expression and purification was performed as described previously [16]. Protein expression was induced with 1 mM IPTG at 25°C for 6 hrs and at 37°C for 3 hrs. Overexpression of the recombinant protein was analyzed in the cell lysate. Purification under native conditions *via *affinity chromatography with Ni-NTA agarose (Qiagen) was performed to assess yields of purified proteins. Cell lysates and purified proteins were analyzed by SDS-PAGE with 15% polyacrylamide separation gels as described [41]. Expression levels in cell lysates and yields of purified proteins were subjectively attributed to one of four categories: 3 (high), 2 (moderate), 1 (low), 0 (not purified).

### Large-scale protein expression and purification

According to protein yields obtained after small-scale expression and purification, culture volumes were adjusted for large-scale expression and purification. Proteins that were purified in high and moderate yields were expressed in 100 ml or 150 ml cultures, respectively. The following expression and purification protocol was applied per 100 ml of bacterial culture: 5 ml pre-culture was grown for 16 hrs at 37°C, followed by the addition of 95 ml of pre-warmed SB medium with ampicillin [16]. At the optical density (OD) of 1.5 at 600 nm, protein expression was induced for 4 hrs at 37°C with 1 mM IPTG or 6 hrs at 25°C. Cells were harvested by centrifugation (4000 × g, 4°C, 15 min) in 50 ml polypropylene tubes (BD Falcon). The cell pellet was frozen in liquid nitrogen and stored at -80°C. To purify protein, the cell pellet was thawed on ice for 15 min and resuspended in 3 ml of lysis buffer [16]. 450 μl of lysozyme solution (1 mg/ml) was added and incubated on ice for 30 min. 600 μl of benzonase buffer (20 mM Tris, pH 8.0, 10 mM MgCl_2_) containing 0.4 unit/μl benzonase grade II (Merck) was added and incubated on ice for 30 min. The lysate was centrifuged at 4900 × g, 4°C, 45 min. The supernatant was transferred to individual cavities of a 24-deepwell plate (Brand) and 600 μl of 50% Ni-NTA agarose (Qiagen) was added. The plate was shaken for 30 min at 10°C and the lysate-bead mixture was transferred to a well of a 96-well filter plate (Macherey & Nagel, No 738655.M). Ni-NTA agarose was washed 6 times with 1.5 ml of washing buffer. Protein was eluted with 600 μl of elution buffer [16]. EDTA (final concentration: 0.5 mM) was added to bind residual Ni^2+ ^ions in the protein solution. In order to exchange the buffer, 500 μl of the protein solution was applied on a NAP-5 column (GE healthcare) and the protein was eluted with 1 ml of phosphate buffered saline (PBS, pH 7.4).

### LPS removal with polymyxin B coated magnetic beads

After changing the buffer, we treated the protein solutions with polymyxin B coated magnetic beads (25 mg/ml; Chemicell, Berlin, Germany) in order to remove LPS. Per protein, 10 mg of LPS removal beads were transferred into 1.5 ml reaction tubes (Eppendorf) and washed three times with 1 ml PBS. To bind LPS, protein solutions were added to the beads and mixed constantly for 30 min at 4°C. Afterwards, the tubes were placed in a magnet and the cleared protein solutions were transferred into fresh reaction tubes.

### Sterilization of protein solutions

Protein solutions were filtered with 0.2 μm syringe filters (Pall). Protein concentrations were determined by photometric determination of the OD at 280 nm. In case of protein loss during downstream applications, such as buffer exchange and LPS removal, proteins were expressed in larger volumes (1–2 l).

### Whole blood stimulations with protein preparations

Aliquots of protein solutions 98, 251, 253, and 256 (see additional file 1: SummaryOfResults.xls) were treated with LPS removal beads. Treated and untreated protein preparations were used for whole blood stimulations as described previously [42]. Per ml of blood, 5 μg of protein was used. Fixed and permeabilized cells were stained with following antibodies (BD): CD4-peridinin chlorophyll A protein (PerCP), CD154 (CD40 Ligand)-phycoerythrin (PE), IFN-γ-allophycocyanin (APC). Cells were analyzed using a FACScalibur flow cytometer and Cell Quest software (both BD). CD4+ T cells were gated electronically and were quantified as the frequencies of cells that were double-positive for CD40 Ligand (CD40L) and IFN-γ. Frequencies resulting from stimulations with treated proteins vs. untreated proteins were related to each other. CD40L was shown to be a universal marker for activation of antigen-specific CD4+ T cells [42]. We used CD40L up-regulation as a marker for T cell activation upon stimulation with our recombinant *E. coli *proteins.

### LAL test

Proteins 251 and 96 were treated with polymyxin B beads and LPS contents in the protein solutions were quantified with the *Limulus *amoebocyte lysate (LAL) test by Profos (Regensburg, Germany).

### Analyses of codon usage and prediction of transmembrane helices

CAI values [25] were determined using the EMBOSS software package (4.0.0) and the codon usage file Eecoli_high.cut [43]; the number of rare codons was established using the NIH Rare Codon Calculator [44]; for prediction of transmembrane helices we used the CBS prediction server TMHMM (2.0) [45].

## Competing interests

The author(s) declare that they have no competing interests.

## Authors' contributions

AE performed the experimental part, analyzed the data and drafted the manuscript.

KB conceived the study, supervised cloning, expression and purification experiments, and revised the manuscript.

JS conceived the study, supervised immunological assays, and revised the manuscript.

AT conceived the study, supervised immunological assays, and revised the manuscript.

RD conceived the study, supervised immunological assays, and revised the manuscript.

TA conceived the study, supervised experiments and edited the manuscript.

All authors have read and approved the manuscript

## Supplementary Material

Additional File 1 Summary Of Results. The table summarizes results of cloning of *E. coli *genes into pQTEV2, expression and yield in small-scale purification, Gateway recombination cloning, small-scale purification, large-scale purification with a minimum of 500 μg (bolt letters), analysis of codon usage (CAI), number of rare codons, and prediction of transmembrane helices. Click here for file
